# Roadmaps Of Resistance: Motorist Greenbook Safe Spaces And Modern-Day Subjective Cognitive Decline

**DOI:** 10.1093/geroni/igaf122.4187

**Published:** 2025-12-31

**Authors:** Jamila Rodriguez, Sydney Moseley, Pedro Pinheiro-Chagas, Muriel Taks Calle, Paris Adkins-Jackson, Tanisha Hill-Jarrett

**Affiliations:** Boston University, Boston, Massachusetts, United States; University of California, San Francisco, San Francisco, California, United States; University of California, San Francisco, San Francisco, California, United States; Columbia University Mailman School of Public Health, New York, New York, United States; Columbia University Mailman School of Public Health, New York, New York, United States; University of California Memory and Aging Center, San Francisco, California, United States

## Abstract

**Introduction:**

The Greenbook is a historic list of safe spaces for individuals racialized as Black living in the U.S. under Jim Crow. The literature on structural racism and poorer cognitive aging across generations suggests the presence of safe spaces (i.e., structural resilience) may mitigate contemporary disparities in cognitive aging, like subjective cognitive decline (SCD). This study examines the state-level association between Greenbook sites and SCD.

**Methods:**

The state-level sum of 1948 Greenbook sites was transformed into quartiles representing sites per square mile (Q1=fewer sites/sq/mi; Q4 = most sites/sq/mi). These data were linked with state-level data from a single-item reporting SCD from 2015/2016 CDC Behavioral Risk Factor Surveillance System (BRFSS) for a sample of older adults (N = 21,378, aged>45). We compared participants across racialized groups and gender living in states with fewer Greenbook sites (Q1,Q2,Q3) to those living in states with more sites (Q4) on modern-day SCD (adjusting for income, degree attainment, and age).

**Results:**

Accounting for covariates, women racialized as Black had the highest odds of reporting SCD (OR: 1.26, 95% C.I. 1.11-1.44) compared to men racialized as White. Across all participants except men racialized as Black, living in states with the fewest Greenbook sites per square mile compared to those in states with the highest associated with modern-day SCD.

**Discussion:**

Living near fewer Greenbook sites is associated with present-day SCD among older adults. This suggests historic safe spaces served as protective factors against SCD. The Greenbook is a refuge from historic structural harms that shape health inequities today.

